# Oral antihypertensive regimens (nifedipine retard, labetalol, and methyldopa) for management of severe hypertension in pregnancy: an open-label, randomised controlled trial

**DOI:** 10.1016/S0140-6736(19)31282-6

**Published:** 2019-09-21

**Authors:** Thomas Easterling, Shuchita Mundle, Hillary Bracken, Seema Parvekar, Sulabha Mool, Laura A Magee, Peter von Dadelszen, Tara Shochet, Beverly Winikoff

**Affiliations:** a Department of Obstetrics and Gynecology, University of Washington, Seattle, WA, USA; b Department of Obstetrics and Gynecology, Government Medical College, Nagpur, India; c Gynuity Health Projects, New York, NY, USA; d Department of Obstetrics and Gynaecology, Daga Memorial Women's Government Hospital, Nagpur, India; e Department of Women and Children's Health, King's College London, London, UK

## Abstract

**Background:**

Hypertension is the most common medical disorder in pregnancy, complicating one in ten pregnancies. Treatment of severely increased blood pressure is widely recommended to reduce the risk for maternal complications. Regimens for the acute treatment of severe hypertension typically include intravenous medications. Although effective, these drugs require venous access and careful fetal monitoring and might not be feasible in busy or low-resource environments. We therefore aimed to compare the efficacy and safety of three oral drugs, labetalol, nifedipine retard, and methyldopa for the management of severe hypertension in pregnancy.

**Methods:**

In this multicentre, parallel-group, open-label, randomised controlled trial, we compared these oral antihypertensives in two public hospitals in Nagpur, India. Pregnant women were eligible for the trial if they were aged at least 18 years; they were pregnant with fetuses that had reached a gestational age of at least 28 weeks; they required pharmacological blood pressure control for severe hypertension (systolic blood pressure ≥160 mm Hg or diastolic blood pressure ≥110 mm Hg); and were able to swallow oral medications. Women were randomly assigned to receive 10 mg oral nifedipine, 200 mg oral labetalol (hourly, in both of which the dose could be escalated if hypertension was maintained), or 1000 mg methyldopa (a single dose, without dose escalation). Masking of participants, study investigators, and care providers to group allocation was not possible because of different escalation protocols in the study groups. The primary outcome was blood pressure control (defined as 120–150 mm Hg systolic blood pressure and 70–100 mm Hg diastolic blood pressure) within 6 h with no adverse outcomes. This study is registered with ClinicalTrials.gov, number NCT01912677, and the Clinical Trial Registry, India, number ctri/2013/08/003866.

**Findings:**

Between April 1, 2015, and Aug 21, 2017, we screened 2307 women for their inclusion in the study. We excluded 1413 (61%) women who were ineligible, declined to participate, had impending eclampsia, were in active labour, or had a combination of these factors. 11 (4%) women in the nifedipine group, ten (3%) women in the labetalol group, and 11 (4%) women in the methyldopa group were ineligible for treatment (because they had only one qualifying blood pressure measurement) or had treatment stopped (because of delivery or transfer elsewhere). 894 (39%) women were randomly assigned to a treatment group and were included in the intention-to-treat analysis: 298 (33%) women were assigned to receive nifedipine, 295 (33%) women were assigned to receive labetalol, and 301 (33%) women were assigned to receive methyldopa. The primary outcome was significantly more common in women in the nifedipine group than in those in the methyldopa group (249 [84%] women *vs* 230 [76%] women; p=0·03). However, the primary outcome did not differ between the nifedipine and labetalol groups (249 [84%] women *vs* 228 [77%] women; p=0·05) or the labetalol and methyldopa groups (p=0·80). Seven serious adverse events (1% of births) were reported during the study: one (<1%) woman in the labetalol group had an intrapartum seizure and six (1%) neonates (one [<1%] neonate in the nifedipine group, two [1%] neonates in the labetalol group, and three [1%] neonates in the methyldopa group) were stillborn. No birth had more than one adverse event.

**Interpretation:**

All oral antihypertensives reduced blood pressure to the reference range in most women. As single drugs, nifedipine retard use resulted in a greater frequency of primary outcome attainment than labetalol or methyldopa use. All three oral drugs—methyldopa, nifedipine, and labetalol—are viable initial options for treating severe hypertension in low-resource settings.

**Funding:**

PREEMPT (University of British Columbia, Vancouver, BC, Canada; grantee of Bill & Melinda Gates Foundation).

## Introduction

Hypertension is the most common medical disorder in pregnancy, and this condition complicates one in ten pregnancies.^1^ Hypertensive disorders of pregnancy include chronic hypertension (ie, hypertension diagnosed before 20 weeks of gestation), pre-eclampsia and eclampsia, chronic hypertension with superimposed pre-eclampsia (a diagnosis of chronic hypertension outside pregnancy or before 20 weeks' gestation and a sudden exacerbation of hypertension or manifestations of end-organ involvement such as new or increased proteinuria, increased liver enzymes, thrombocytopenia, pulmonary oedema, renal insufficiency, or symptoms such as severe headache or right upper-quadrant pain), and gestational hypertension. Hypertension in pregnancy is associated with adverse effects for the mother and baby, including fetal growth restriction, preterm delivery, and maternal, fetal, and neonatal morbidity and mortality.^1^ A 2013 WHO international hospital survey^2^ on maternal and neonatal health found an incidence of pre-eclampsia of 2·5% and an incidence of eclampsia of 0·3% in 314 623 women from Asia, Africa, and Latin America. Women with hypertensive disorders of pregnancy are also at greater risk for the development of cardiovascular risk factors (hypertension, type 2 diabetes, and obesity), chronic kidney disease, premature cardiovascular disease (cardiac, cerebrovascular, and peripheral arterial), and cardiovascular mortality.^3^

**Research in context**

**Evidence before this study**

Before designing our study, we searched PubMed and the Cochrane Database of Clinical Trials with the search terms “oral antihypertensives”, “severe hypertension in pregnancy”, “nifedipine”, “methyldopa”, and “labetalol”. We used MeSH terms and appropriate variations to search for papers published from Jan 1, 1980, to July 31, 2013, without language restrictions. Standard Cochrane methods were used to assess quality. In 2014, two of the authors (PvD, LAM) published a systematic review of randomised controlled trials that featured at least one group who were treated with a single oral antihypertensive drug in pregnancy and the post-partum period, to reduce systolic blood pressure measurements of at least 160 mm Hg, diastolic blood pressures of at least 110 mm Hg, or both. This systematic review identified 14 studies in pregnancy. Most trials compared oral or sublingual nifedipine capsules with another drug, usually parenteral hydralazine or labetalol. A 2018 network meta-analysis suggested a similar efficacy between nifedipine, intravenous hydralazine, and intravenous labetalol in the treatment of severe hypertension in pregnancy. We found no studies that directly compared the three most commonly used oral antihypertensives: labetalol, methyldopa, and nifedipine. A Cochrane review of drugs for treatment of very high blood pressure during pregnancy also found insufficient data to recommend a specific drug, and it concluded that the choice of antihypertensive should depend on clinicians' experience and familiarity with the drug, known adverse effects, and women's experiences. WHO recommends the use of antihypertensive drug for treatment of severe hypertension in pregnancy, but it does not recommend a specific oral drug.

**Added value of this study**

Our study directly compares three commonly used oral antihypertensive drugs that are recommended for treatment of severe hypertension in pregnancy by WHO: nifedipine, labetalol, and methyldopa. Our findings show that, for pregnant women with severe hypertension, oral nifedipine retard was more effective than methyldopa at achieving a primary outcome of blood pressure control without adverse events within 6 h when additional medications were used. Oral nifedipine retard and labetalol, as single drugs, were significantly more effective than methyldopa. The frequency of primary outcome attainment was high and maternal adverse events were low in all three treatment groups. However, more neonates born to women assigned to the nifedipine group were admitted to the intensive care unit, primarily because more low or very low birthweight babies were born to mothers in the nifedipine group.

**Implications of all the available evidence**

Our study provides additional information on the relative effectiveness of methyldopa, an oral medication widely available in many settings. The results suggest that all three oral drugs—methyldopa, nifedipine, and labetalol—are viable initial options for treating severe hypertension in pregnancy in low-resource settings. Our findings provide important reassurance for use of drugs available in many settings, especially given the wide variability of availability of oral antihypertensive medications in low-income and middle-income countries. Our findings also provide a rationale for a structured approach to the use of oral antihypertensive medications in a broad spectrum of medical settings, such that delays in treatment can be reduced. Our study results also suggest a need to expand access to and use of oral antihypertensive drugs for treatment of severe hypertension. WHO has listed only intravenous hydralazine and methyldopa in the latest essential drugs list for treating severe hypertension in pregnancy; nifedipine is only included as a treatment for preterm birth. In our study, only one patient received additional treatment with an intravenous medication. Methyldopa, the drug found to be least effective in this study, might be the only drug available in some settings. Based on this new evidence, efforts should be made to include nifedipine or labetalol on the essential drugs list for treatment of severe hypertension in pregnancy, especially given its restricted use as a treatment for preterm birth.

Severe hypertension in pregnancy is defined by a systolic blood pressure of at least 160 mm Hg or diastolic blood pressure of at least 110 mm Hg, and either of these clinical signs is considered to be an obstetric emergency for both mother and fetus; urgent antihypertensive treatment is recommended.^4^, ^5^, ^6^, ^7^ Management of severe hypertension primarily aims to reduce the risk of cardiovascular or cerebrovascular events. There is no consensus on the relative efficacy and safety of the medications to treat severe hypertension in pregnancy, and the most recent Cochrane review^7^ found insufficient data to recommend a specific drug, concluding that the choice of antihypertensive should be guided by clinicians' experience and familiarity with the drug, known adverse effects, and women's experiences.

Clinical trials have typically evaluated medications that are administered intravenously (such as hydralazine or labetalol).^8^ Although these regimens are effective in lowering blood pressure, they require intravenous access, have the potential to reduce blood pressure precipitously, and require careful fetal monitoring. However, oral medications can be provided in several health-care settings, do not require cold storage, do not require special equipment and a provider trained in intravenous drug administration, and are available in most low-income and middle-income countries.^9^

Three oral antihypertensive drugs (nifedipine [a calcium-channel blocker], labetalol [a combined α blocker and β blocker], and methyldopa [a CNS α agonist]) have been used extensively in pregnancy and have shown a low incidence of medical complications. Oral regimens have been suggested and a few have been described in clinical practice.^10^, ^11^ Five trials^12^, ^13^, ^14^, ^15^, ^16^ of oral antihypertensive therapy for severe hypertension in pregnancy have been conducted, including trials of these three drugs.

Although oral drugs appear to be optimal with regard to ease of storage and administration, there are few direct comparisons of the three oral drugs recommended for management of acute severe hypertension in pregnancy in low-resource settings by WHO. We therefore aimed to compare the efficacy and safety of oral labetalol, nifedipine retard, and methyldopa for the management of severe hypertension in pregnancy.

## Methods

### Study design and participants

In this multicentre, parallel-group, open-label, randomised controlled trial, we compared these oral antihypertensives in two public hospitals in Nagpur, India: the Government Medical College, Nagpur and Daga Memorial Women's Government Hospital. The Government Medical College, Nagpur, is a university hospital and tertiary referral centre that is responsible for about 13 000 deliveries annually; this hospital reports roughly an 8% incidence of severe pre-eclampsia and a 1·7% incidence of eclampsia. Daga is a district-level hospital that provides basic and emergency obstetric care, is responsible for about 15 000 deliveries annually, and it has a blood bank, but it does not have the capacity to deal with complicated pregnancies and severely ill women. Such women are generally referred to Government Medical College, Nagpur.

Pregnant women were eligible for the trial if they were aged at least 18 years; they were pregnant with fetuses that had reached a gestational age of at least 28 weeks; they required pharmacological blood pressure control for severe hypertension, defined as a systolic blood pressure of at least 160 mm Hg or a diastolic blood pressure of at least 110 mm Hg (when measured twice, 15 min apart, with the woman sitting quietly for several minutes with the arm cuff at heart level, and the diastolic blood pressure designated as the fifth Korotkoff sound by the mercury sphygmomanometer); and were able to swallow oral medications. Women who were unable to give consent; who had an indication for an emergency caesarean section or a known fetal anomaly; who had received antihypertensive medication in the previous 12 h; who were actively wheezing; or who had a history of asthma complications, known coronary artery disease, type 1 diabetes with microvascular complications, signs of heart failure, or clinical dissection of the aorta were ineligible. Women were screened for participation in the study by their doctors when the need for pharmacological treatment of blood pressure was detected in the antenatal clinic or labour ward, either in routine appointments or in secondary care after presenting with signs and symptoms. Eligible women were enrolled by labour ward research staff.

All enrolled women provided informed written consent. The study was approved by the Institutional Ethics Committees at Government Medical College, Nagpur (no. 430EC/Pharmac/GMC/NGP/2013), the NKP Salve Institute of Medical Sciences and Lata Mangeshkar Hospital in Nagpur (Pharmac/82/2013), the Indian Council of Medical Research (5/7/1026/13-RCH), and the Drug Controller General of India (CT/17/14-DCG [I]). A copy of the protocol is available online.

### Randomisation and masking

We randomly assigned women to receive nifedipine, labetalol, or methyldopa. This assignment was done after the women gave informed consent to participate. The enrolling research staff opened a sequentially numbered, sealed, opaque envelope that contained the participant's assigned group. These envelopes were generated by Gynuity Health Projects staff with a randomisation code that was based on a computerised pseudorandom number generator. Randomisation was stratified by centre and used block sizes of nine and ten. Masking of participants, study investigators, and care providers to group allocation was not possible given differences in dose escalation protocols in the three study groups.

### Procedures

Women who were randomly assigned to receive nifedipine were given an initial dose of 10 mg oral nifedipine (10 mg Nicardia retard [JB Chemicals and Pharmaceuticals, Mumbai, India]); if their systolic blood pressure exceeded 155 mm Hg or their diastolic blood pressure exceeded 105 mm Hg after 1 h, an additional 10 mg dose could be provided each hour for two additional doses (to a total of 30 mg). Women who were randomly assigned to receive labetalol were given an initial dose of 200 mg oral labetalol (100 mg Labetet [Sun Pharma Laboratories, Mumbai, India]); if their systolic blood pressure exceeded 155 mm Hg or their diastolic blood pressure exceeded 105 mm Hg after 1 h, an additional 200 mg dose could be provided each hour for two additional doses (to a total of 600 mg). Women who were randomly assigned to receive methyldopa were given a single dose of 1000 mg methyldopa (500 mg Alphadopa [Wockhardt, Mumbai, India]), without dose escalation for the 6-h study period.

For women in all three groups, we monitored and recorded pulse and blood pressure every 20 min with an automatic digital blood pressure cuff. The blood pressure measurements were confirmed with a mercury sphygmomanometer whenever an automated reading indicated a trial-qualifying blood pressure. Oxygen saturation was measured and recorded at baseline and at 2 h, 4 h and 6 h. The Glasgow Coma Scale assessment was administered by a research clinician at baseline, 2 h, and 6 h. Aspartate transaminase, platelet count, and serum creatinine concentration were measured at baseline and 6 h. Maternal side-effects were assessed at baseline, 2 h, and 6 h. The fetal heart rate was monitored at baseline and 6 h.

Magnesium sulphate was administered to participants with severe pre-eclampsia as per hospital protocols at provider discretion. Participant data, including demographic characteristics, medical and pregnancy history, and labour course and outcomes, were collected by the research staff.

We interviewed women after the study, after they were stable and before discharge from the study hospital. They were asked about their experience with pain, side-effects, and the acceptability of the treatment regimen.

### Outcomes

The primary outcome was blood pressure control (defined as 120–150 mm Hg systolic blood pressure and 70–100 mm Hg diastolic blood pressure) within 6 h with no adverse outcomes. These adverse outcomes included hypotension (systolic blood pressure <120 mm Hg, diastolic blood pressure <70 mm Hg, or both, and fetal distress), caesarean section for fetal distress up to 2 h after the end of the study period, severe headache, severe headache requiring discontinuation of drug, or eclampsia. The secondary outcomes were the need to change drug regimen or provide additional medications; placental abruption; maternal side-effects associated with worsening maternal pre-eclampsia (including chest pain, dyspnoea, headache that resulted in change in treatment, visual symptoms such as flashes or diplopia, epigastric or right upper quadrant abdominal pain, and nausea or vomiting); maternal morbidity (eclampsia or seizure, adverse CNS outcomes such as stroke or cortical blindness, HELLP syndrome, pulmonary oedema [indicated by oxygen saturation <90% and abnormal chest x-ray], oliguria [<25 mL/h for 2 h], and disseminated intravascular coagulation); maternal death; caesarean delivery; and enrolment-to-delivery interval.

As prespecified maternal endpoints, we also assessed the duration of hospital stay, admission to an intensive care unit or high-dependency unit, and any use of dialysis or mechnical ventilation. We also assessed fetal or neonatal complications, including neonatal morbidity (respiratory distress syndrome that required oxygen supplementation, abnormal cerebral ultrasound, convulsions, bradycardia [ie, sustained heart rate <100 bpm] beyond resuscitation and requiring intervention); neonatal admission to an intensive care unit, including the duration of intensive care unit admission, oxygen use, and mechanical ventilation; stillbirth; and neonatal death.

### Statistical analysis

The sample size was estimated a priori on the basis of the primary outcome, assuming a prevalence of 75% with nifedipine, 62·5% with labetalol, and 50% with methyldopa, based on published data.^16^ The participants in the nifedipine group served as the comparison group for both the labetalol and methyldopa regimens; the comparison of labetalol and nifedipine required a sample size of 261 patients in each group, assuming a two-tailed test, α=0·05 and a power of 80%, and a simple Bonferroni correction. Under the same statistical assumptions, with a nifedipine sample size of 261 patients, the methyldopa group required 59 patients. To account for loss to follow-up, the initial sample size included 671 women (nifedipine, 298 women; labetalol, 298 women; methyldopa, 75 women). In October, 2015, after 6 months of enrolment, the Trial Steering Committee requested an increase in size of the methyldopa group because a large cohort study^17^ suggested that the drug was more effective than originally estimated. The Trial Steering Committee believed that the original sample size would be underpowered to provide useful evidence regarding methyldopa's effectiveness. Thus, the sample size in the methyldopa arm increased from 75 women to 298 women. The new sample size thus included 298 women in each treatment group. In January, 2016, we implemented a new randomisation sequence, resulting in a change in the enrolment ratio (nifedipine: labetalol: methyldopa) during the two periods: from 4:4:1 in phase 1, comprising patients 1–265, to 3:3:4 in phase 2, comprising patients 266–894.

The Data and Safety Monitoring Board did one planned interim analysis after half of the total women were enrolled (n=447) with blood pressure control within 6 h without an adverse outcome as the primary outcome, and they concluded that the trial should continue.

All analyses were by intention to treat. Generalised linear modelling (regression) methods were used where possible to estimate effect sizes with their corresponding 95% CIs. Binomial regression was used for the primary outcome measure and other binary categorical measures, whereas multinomial regression was used for multi-category variables; Fisher exact tests were used in the presence of zero or very small frequency counts. Continuous measures were analysed by use of linear regression with mean differences or by Mann–Whitney *U* tests with median differences, according to their distributions. Analyses were done with Stata/SE version 12.1. Statistical significance was set at 5% for primary outcomes and at 1% for secondary outcomes.

The study was monitored by independent data monitoring committees, and it is registered with ClinicalTrials.gov (NCT01912677) and the Clinical Trial Registry, India (ctri/2013/08/003866).

### Role of the funding source

The funder of the study had no role in study design, data collection, data analysis, data interpretation, or writing of the report. The corresponding author had full access to all the data in the study and had final responsibility for the decision to submit for publication.

## Results

Between April 1, 2015, and Aug 21, 2017, we screened 2307 women at the two study sites for their inclusion in the study. We excluded 1413 (61%) women who were ineligible, declined to participate, had impending eclampsia, were in active labour, or had a combination of these factors (figure). Women with impending eclampsia or in active labour were excluded by enrolling providers because they required immediate delivery, and it was therefore assumed that they would not complete the 6-h study period. 894 (39%) women were randomly assigned to a treatment group and were included in the intention-to-treat analysis: 298 (33%) women were assigned to receive nifedipine, 295 (33%) women were assigned to receive labetalol, and 301 (33%) women were assigned to receive methyldopa. Of those randomised, three (1%) women in the nifedipine group, one (<1%) woman in the labetalol group, and three (1%) women in the methyldopa group only had one qualifying blood pressure measurement (ie, a systolic blood pressure measurement of ≥160 mm Hg or a diastolic blood pressure measurement of ≥110 mm Hg) at the time of enrolment and were therefore ineligible for treatment. Eight (3%) women in the nifedipine group, nine (3%) women in the labetalol group, and eight (3%) women in the methyldopa group stopped treatment before the end of the study period (6 h) because of delivery or transfer to another facility. For these women, we used the last blood pressure recorded before transfer or delivery for the primary outcome analysis. We were missing data on the mode or outcome of delivery for 14 women (three [1%] women in the nifedipine group, five [2%] women in the labetalol group, and six [2%] women in the methyldopa group) because they were discharged from the hospital before delivery or ultimately gave birth at another facility.

**Figure:**
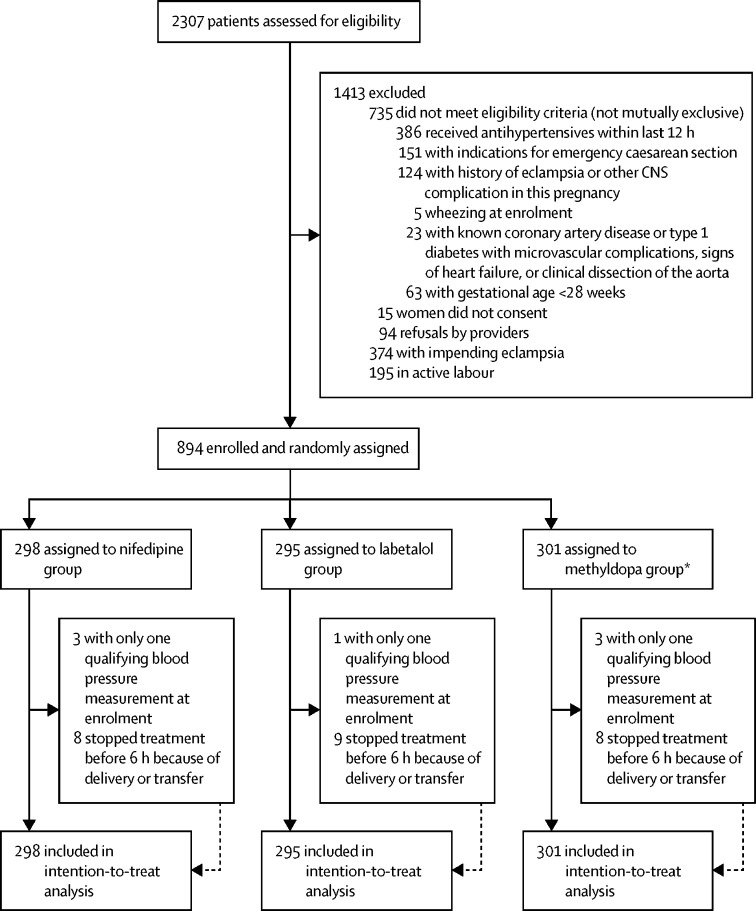
Trial profile *After 6 months of enrolment, the Trial Steering Committee requested an increase in the number of participants in the methyldopa group, because a large cohort study suggested the drug was more effective than originally estimated.^17^ Thus, we increased the number in the methyldopa group from 75 women to 298 women.

The baseline characteristics of the women in the three groups were similar, with no notable differences across the groups (table 1). At enrolment, the mean highest systolic blood pressure measurement was 160 mm Hg (SD 11·5) and the mean highest diastolic blood pressure measurement was 110 mm Hg (SD 6·8). Approximately one third of women had proteinuria of 2+ or more at enrolment. However, only 38 (4%) of 894 women received magnesium sulphate therapy in the 12 h before enrolment. 189 (21%) women had received an antihypertensive drug at least 12 h before enrolment. The median time from randomisation to the start of oral antihypertensive therapy was 10 min in all groups, with no delay of more than 77 min (table 2). All enrolled women received at least one dose of their allocated medication.

**Table 1:** Baseline characteristics

		**Nifedipine (n=298)**	**Labetalol (n=295)**	**Methyldopa (n=301)**
**Study site**				
Government Medical College		150 (50%)	151 (51%)	151 (50%)
Daga Women's Hospital		148 (50%)	144 (49%)	150 (50%)
**Maternal demographics**				
Maternal age, years		25·6 (4·0)	25·5 (4·2)	25·5 (4·2)
Body-mass index, kg/m^2^		27·1 (4·3; 16·4–43·2)	27·4 (4·3; 17·8–43·7)	27·3 (4·3; 16·2–39·2)
**Pregnancy characteristics**				
Gestational age, weeks		36·5 (2·9)	36·5 (2·7)	36·7 (2·6)
Multiple pregnancy		12 (4%)	7 (2%)	9 (3%)
Fetus alive at enrolment		298 (100%)	295 (100%)	301 (100%)
**Clinical measurements**				
Mean systolic blood pressure, mm Hg		158 (11·1; 130–200)	158 (11·5; 130–200)	157 (11·7; 130–200)
Highest systolic blood pressure at enrolment, mm Hg*				
	<160	109 (37%)	102 (35%)	109 (36%)
	160–169	112 (38%)	125 (42%)	129 (43%)
	≥170	77 (26%)	68 (23%)	63 (21%)
Mean diastolic blood pressure, mm Hg		109 (7·6; 87–165)	108 (7·1; 87–140)	108 (6·8; 90–130)
Highest diastolic blood pressure at enrolment, mm Hg				
	<110	56 (19%)	64 (22%)	73 (24%)
	110–119	202 (68%)	186 (63%)	199 (66%)
	≥120	40 (13%)	45 (15%)	29 (10%)
Urinary dipstick proteinuria				
	Nil or trace	108 (36%)	98 (33%)	105 (35%)
	1+	93 (31%)	91 (31%)	104 (35%)
	2+	69 (23%)	70 (24%)	60 (20%)
	>3+	28 (9%)	36 (12%)	32 (11%)
Oxygen saturation <95%		0	1 (<1%)	0
Mean heart rate, bpm		95·7 (13·9; 55–141)	97·4 (12·6; 62–134)	97·2 (14·8; 56–142)
Source of admission				
	Referred	79 (27%)	83 (28%)	100 (33%)
	Outpatient department	180 (60%)	179 (61%)	173 (58%)
	Walk-in	39 (13%)	33 (11%)	28 (9%)
Received magnesium sulphate in 12 h before enrolment		16 (5%)	7 (2%)	15 (5%)
Received an antihypertensive drug at least 12 h before enrolment		63 (21%)	65 (22%)	61 (20%)
	Methyldopa	12 (4%)	16 (5%)	13 (4%)
	Nifedipine	35 (12%)	30 (10%)	29 (10%)
	Labetalol	21 (7%)	22 (8%)	20 (7%)
Plans for delivery at time of enrolment				
	Planned induction of labour	118 (40%)	128 (43%)	114 (38%)
	Planned expectant management	180 (60%)	167 (57%)	187 (62%)
**Laboratory values, n (%)/N**				
Platelet count <1 × 10^5^/L		16 (6%)/287	12 (4%)/286	17 (6%)/295
Serum creatinine ≥1·0		22 (8%)/294	23 (8%)/294	20 (7%)/300
Aspartate transaminase >80 IU/L		10 (3%)/293	12 (4%)/293	20 (7%)/301
Aspartate transaminase >80 IU/L and platelet count <1 × 10^5^/L		1 (<1%)/285	1 (<1%)/285	3 (1%)/295

Data are n (%), n (%)/N, mean (SD), or mean (SD; range).

* Seven women did not meet blood pressure eligibility criteria at the time of enrolment: three women in the nifedipine group, one woman in the labetalol group, and three women in the methyldopa group.

**Table 2:** Study completion and adherence to treatment

		**Nifedipine (n=298)**	**Labetalol (n=295)**	**Methyldopa (n=301)**	**Absolute difference, nifedipine *vs* labetalol (95% CI)**	**Absolute difference, nifedipine *vs* methyldopa (95% CI)**	**Absolute difference, labetalol *vs* methyldopa (95% CI)**
Median time from randomisation to start of treatment, min		10 (0 to 55)	10 (0 to 77)	10 (0 to 45)	0 (−1·7 to 1·7)	0 (−1·7 to 1·7)	0 (−1·7 to 1·7)
Transferred to another site or delivered before 6 h		8 (3%)	9 (3%)	8 (3%)	−0·4 (−3·1 to 2·3)	0·03 (−2·6 to 2·6)	0·4 (−2·3 to 3·1)
Received at least one dose of allocated medication		298 (100%)	295 (100%)	301 (100%)	..	..	..
Received a second dose of allocated medication		131 (44%)	142 (48%)	0	−4·1 (−12·1 to 3·9)*	..	..
	Median time received after randomisation, min	85 (60 to 365)	130 (60 to 370)	0	−45 (−78 to −12)	..	..
Received a third dose of allocated medication		45 (15%)	64 (22%)	0	−6·6 (−12·8 to 0·4)†	..	..
	Median time received after randomisation, min	175 (120 to 360)	190 (125 to 330)	0	−15 (−66 to 36)	..	..
Received another antihypertensive drug during the study period‡		2 (1%)	9 (3%)	56 (19%)	−2·4 (−4·6 to −0·2)	−17·9 (−22·4 to −13·4)	−15·6 (−20·4 to 10·7)
	Nifedipine§	0	8 (3%)	47 (16%)	−2·7 (−4·5 to −0·9)	−15·6 (−19·7 to −11·5)	−12·9 (−17·4 to −8·4)
	Labetalol (orally)¶	1 (<1%)	0	10 (3%)	0·3 (−0·3 to 0·9)	−3·0 (−5·1 to −0·9)	−3·3 (−5·3 to −1·3)
	Labetalol (intravenously)	0	1 (<1%)	0	−0·3 (−0·9 to 0·3)	..	0·3 (−0·3 to 0·9)
	Methyldopa	1 (<1%)	0	1 (<1%)	0·3 (−0·3 to 0·9)	0·003 (−0·9 to 0·9)	−0·3 (−0·9 to 0·3)

Data are median (range) or n (%), unless otherwise indicated. In the methyldopa group, one woman received a dose each of nifedipine and labetalol, one woman received a dose of nifedipine and an additional methyldopa dose, and four women received two doses of nifedipine.

* p=0·01.

† p=0·04.

‡ Nifedipine *vs* labetalol: p=0·04; nifedipine *vs* methyldopa: p<0·0001; labetalol *vs* methyldopa: p<0·0001.

§ Nifedipine *vs* labetalol: p=0·004; nifedipine *vs* methyldopa: p<0·0001; labetalol *vs* methyldopa: p=0·002.

¶ Nifedipine *vs* methyldopa: p=0·01; labetalol *vs* methyldopa: p=0·01.

The primary outcome—blood pressure control within the 6-h study period, with no adverse outcomes—was significantly more common in women in the nifedipine group than in those in the methyldopa group (249 [84%] women *vs* 230 [76%] women; p=0·03; table 3). However, the primary outcome did not differ between the nifedipine and labetalol groups (248 [84%] women *vs* 228 [77%] women; p=0·05) or the labetalol and methyldopa groups (p=0·80).

**Table 3:** Maternal outcomes

		**Nifedipine (n=298)**	**Labetalol (n=295)**	**Methyldopa (n=301)**	**Absolute difference, nifedipine *vs* labetalol (95% CI)**	**Absolute difference, nifedipine *vs* methyldopa (95% CI)**	**Absolute difference, labetalol *vs* methyldopa (95% CI)**
Primary outcome*							
Achieved primary outcome		249 (84%)	228 (77%)	230 (76%)	6·3 (−0·1 to 12·6)	7·1 (0·8 to 13·5)	0·9 (−5·9 to 7·6)
Exploratory results of primary outcome							
	Achieved primary outcome without needing additional antihypertensive therapy†	247 (83%)	227 (77%)	190 (63%)	6·0 (−0·4 to 12·4)	19·8 (12·9 to 26·7)	13·8 (6·5 to 21·1)
	Reached the blood pressure target	254 (85%)	231 (78%)	232 (77%)	6·9 (0·7 to 13·1)	8·2 (1·9 to 14·4)	1·2 (−5·5 to 7·9)
	Any adverse outcome‡	7 (2%)	4 (1%)	3 (1%)	1·0 (−1·2 to 3·2)	1·4 (−0·7 to 3·4)	0·4 (−1·4 to 2·1)
	Received additional antihypertensive drugs	2 (1%)	9 (3%)	56 (19%)	−2·4 (−4·6 to −0·2)	−17·9 (−22·4 to −13·4)	−15·6 (−20·4 to −10·7)
	Achieved primary outcome at 3 h	219 (74%)	212 (72%)	185 (62%)	1·6 (−5·5 to 8·8)	12·0 (4·6 to 19·5)	10·4 (2·9 to 17·9)
	Received magnesium sulphate during study period	31 (10%)	40 (14%)	34 (11%)	−3·2 (−8·4 to 2·1)	−0·9 (−5·9 to 4·1)	2·3 (−3·0 to 7·6)
**Delivery outcomes**							
Mode of delivery (n=295 *vs* n=290 *vs* n=295)							
	Vaginal delivery	104 (35%)	104 (36%)	116 (39%)	−0·6 (−8·4 to 7·2)	−4·0 (−11·8 to 3·8)	−3·4 (−11·2 to 4·4)
	Forceps delivery	1 (<1%)	0	0	0·3 (−0·3 to 0·9)	0·3 (−0·3 to 0·9)	0 (0 to 0)
	Caesarean section	190 (64%)	186 (64%)	179 (61%)	0·3 (−7·5 to 8·1)	3·7 (−4·1 to 11·5)	3·4 (−4·4 to 11·2)
Indications for caesarean section (n=188 *vs* n=179 *vs* n=178)§							
	Breech presentation	1 (1%)	0	2 (1%)	0·5 (−0·5 to 1·5)	−0·6 (−2·4 to 1·2)	−1·1 (−2·6 to 0·4)
	Twins	2 (1%)	0	0	1·1 (−0·4 to 2·6)	1·1 (−0·4 to 2·6)	0 (0 to 0)
	Fetal heart rate abnormalities	25 (13%)	24 (13%)	22 (12%)	−0·1 (−7·1 to 6·9)	0·9 (−6·0 to 7·8)	1·0 (−6·0 to 8·0)
	Other fetal indications	3 (2%)	3 (2%)	4 (2%)	−0·1 (−2·7 to 2·5)	−0·6 (−3·4 to 2·2)	−0·5 (−3·4 to 2·4)
	Uncontrolled blood pressure	4 (2%)	13 (7%)	10 (6%)	−5·2 (−9·5 to −0·9)	−3·5 (−7·5 to 0·5)	1·7 (−3·4 to 6·8)
	Previous caesarean section	24 (13%)	27 (15%)	21 (12%)	−2·3 (−9·4 to 4·8)	1·0 (−5·7 to 7·7)	3·3 (−3·8 to 10·4)
	Unfavourable cervix	20 (11%)	29 (16%)	22 (12%)	−5·6 (−12·6 to 1·4)	−1·8 (−8·3 to 4·7)	3·8 (−3·4 to 11·1)
	Failed induction of labour	66 (35%)	61 (34%)	62 (35%)	1·0 (−8·7 to 10·7)	0·3 (−9·5 to 10·1)	−0·7 (−10·6 to 9·2)
	Failure to progress after 6-cm dilation	14 (7%)	5 (3%)	7 (4%)	4·6 (0·1 to 9·1)	3·5 (−1·2 to 8·2)	−1·1 (−4·8 to 2·6)
	Other	10 (5%)	5 (3%)	10 (6%)	2·5 (−1·5 to 6·5)	−0·3 (−5·0 to 4·4)	−2·8 (−7·0 to 1·4)
Median time from randomisation to delivery (IQR), h (n=295 *vs* n=290 *vs* n=295)		24·5 (14·5–49·4)	23·6 (14·3–44·7)	22·8 (13·5–46·1)	0·9 (−2·8 to 4·5)	1·8 (−1·7 to 5·2)	0·9 (−2·5 to 4·3)
**Adverse maternal outcomes**¶							
Seizure		0	1 (<1%)	0	−0·3 (−0·9 to 0·3)	0 (0 to 0)	0·3 (−0·3 to 0·9)
Adverse CNS outcome (stroke or cortical blindness)		0	0	0	..	..	..
Pulmonary oedema (oxygen saturation <90% and abnormal chest x-ray)		0	0	0	..	..	..
Oliguria (<25 cm^3^/h for 2 h) up to 2 h after end of study period		0	0	0	..	..	..
Disseminated intravascular coagulation, diagnosed by treating physician		0	0	0	..	..	..
Admission to intensive care unit		0	0	0	..	..	..
Dialysis		0	0	0	..	..	..
Mechanical ventilation		0	0	0	..	..	..
Complications of labour and delivery							
	Placental abruption	0	1 (<1%)	0	−0·3 (−0·9 to 0·3)	0 (0 to 0)	0·2 (−0·5 to 0·9)
	Post-partum haemorrhage	2 (1%)	1 (<1%)	0	0·4 (−0·7 to 1·5)	0·7 (−0·2 to 1·6)	0·2 (−0·5 to 0·9)
	Received blood products after trial entry	10 (3%)	5 (2%)	3 (1%)	1·7 (−0·8 to 4·2)	2·4 (0·1 to 4·7)	0·7 (−1·2 to 2·6)
Maternal death		0	0	0	0 (0 to 0)	0 (0 to 0)	0 (0 to 0)

Data are n (%), unless otherwise indicated.

* Defined as attaining the blood pressure target (120–150 mm Hg systolic and 70–100 mm Hg diastolic) after 6 h without an adverse outcome (systolic blood pressure <120 mm Hg, diastolic blood pressure <70 mm Hg, or both; fetal compromise; or caesarean section for fetal distress, severe headache, or eclampsia) during the study period.

† During the 6-h study period or, if delivered during the study period, last blood pressure measurement before birth.

‡ Included low blood pressure and fetal compromise (nifedipine, n=2; labetalol, n=0; methyldopa, n=0); caesarean section for fetal distress (nifedipine, n=3; labetalol, n=1; methyldopa, n=1); severe headache (defined as a pain score ≥5 on a 7-point visual analogue scale) during or up to 2 h after the end of the study period that resulted in a change in treatment (nifedipine, n=2; labetalol, n=2; methyldopa, n=2); or a seizure during or up to 2 h after the end of the study period (nifedipine, n=0; labetalol, n=1; methyldopa, n=0).

§ Women could have more than one indication.

¶ Between study start and discharge, unless otherwise indicated.

Slightly less than half of women in the nifedipine and labetalol groups received a second dose of their allocated medication, after median times of 85 min (nifedipine) and 130 min (labetalol; p=0·01; table 2). Women in the labetalol group more frequently received a third dose of study treatment than those in the nifedipine group (45 [15%] women *vs* 64 [22%] women; p=0·04). Women assigned to receive methyldopa were more likely to receive an additional or second hypertensive drug during the study period than those receiving nifedipine or labetalol (56 [19%] women *vs* two [1%] women, and *vs* nine [3%] women; p<0·0001 for both); the most common second medication in the methyldopa group was nifedipine. In almost all women administered another hypertensive drug (65 [97%] of 67 women), their blood pressure was more than the target range at the time of administration of the second medication. Only one participant received an intravenous medication (labetalol).

Placental abruption only occurred in one (<1%) woman, in the labetalol group (table 3; appendix). Women in the nifedipine group more frequently reported a headache after 2 h than women assigned to receive either labetalol or methyldopa (15% *vs* 10% *vs* 6%; p=0·03 and p<0·001), which persisted until the end of the study. Headaches were reported to be of similar severity between groups.

Women assigned to receive nifedipine more frequently showed tachycardia (ie, a heart rate of 115 bpm) at one or more monitoring visits than women receiving either labetalol or methyldopa (31% *vs* 14% *vs* 17%; p<0·0001 for both; appendix). Glasgow Coma Scale scores did not vary between groups. One (<1%) woman in the labetalol group had an intrapartum seizure; she convulsed despite receiving magnesium sulphate. The reported acceptability of side-effects did not significantly differ between the three treatment groups. No women died during the study period.

Labour and delivery outcomes did not vary between groups. Approximately two-thirds of women in each group delivered by caesarean section (190 [64%] of 295 women in the nifedipine group; 186 [64%] of 290 women in the labetalol group; and 179 [61%] of 295 women in the methyldopa group), predominantly because of failed inductions of labour and fetal heart rate abnormalities (table 3). The median time from enrolment to delivery was approximately 24 h.

The incidence of stillbirth, neonatal death, and neonatal morbidities did not vary between groups (table 4). However, the frequency of neonatal admission to an intensive care unit was significantly higher in babies born to women assigned to nifedipine versus labetalol (p=0·009) and methyldopa (p=0·004), predominantly because of low or very low birthweight. The mean durations of stay in intensive care units (less than *vs* at least 24 h) did not differ between groups.

**Table 4:** Neonatal outcomes

		**Nifedipine (n=298)**	**Labetalol (n=295)**	**Methyldopa (n=301)**	**Absolute difference, nifedipine *vs* labetalol (95% CI)**	**Absolute difference, nifedipine *vs* methyldopa (95% CI)**	**Absolute difference, labetalol *vs* methyldopa (95% CI)**
**Birth characteristics**							
Gestational age at delivery, weeks (n=305 *vs* n=295 *vs* n=302)		36·9 (2·8; 28·7–40·8)	36·9 (2·5; 29·2–41·1)	37·1 (2·5; 29·3–41·1)	−0·003 (−0·4 to 0·4)	−0·2 (−0·6 to 0·2)	−0·2 (−0·6 to 0·2)
Outcome of delivery							
	Livebirth	299 (97%)	290 (96%)	295 (95%)	0·5 (−2·5 to 3·5)	1·6 (−1·6 to 4·8)	1·1 (−2·2 to 4·4)
	Stillborn	8 (3%)	7 (2%)	8 (3%)	0·3 (−2·1 to 2·7)	0·0 (−2·5 to 2·5)	−0·3 (−2·7 to 2·1)
	Unknown*	3 (1%)	5 (2%)	8 (3%)	−0·7 (−2·5 to 1·1)	−1·6 (−3·7 to 0·5)	−0·9 (−3·2 to 1·4)
Birthweight, g (n=307 *vs* n=297 *vs* n=303)		2300 (661; 574–4200)	2366 (623; 900–4100)	2383 (637; 650–3700)	−65·1 (−167·8 to 37·7)	−82·8 (−186·0 to 20·4)	−17·7 (−118·8 to 83·4)
**Neonatal outcomes among known livebirths**							
Neonatal morbidity							
	Apgar score <7 at 5 min	1 (<1%)/297	2 (1%)/280	1 (<1%)/298	−0·4 (−1·6 to 0·8)	0·0 (−0·9 to 0·9)	0·4 (−0·8 to 1·6)
	Intubated at place of delivery	0/297	0/280	1 (<1%)/298	0 (0 to 0)	−0·3 (−0·9 to 0·3)	−0·3 (−0·9 to 0·3)
	Neonatal convulsions	1 (<1%)/298	2 (1%)/280	0/298	−0·4 (−1·6 to 0·8)	0·3 (−0·3 to 0·9)	0·7 (−0·3 to 1·7)
	Abnormal cerebral ultrasound	0/298	0/280	0/298	0 (0 to 0)	0 (0 to 0)	0 (0 to 0)
	Septicaemia	3 (1%)/297	3 (1%)/280	3 (1%)/298	−0·1 (−1·8 to 1·6)	0·0 (−1·6 to 1·6)	0·1 (−1·6 to 1·8)
	Bradycardia (heart rate <110 bpm)	0/297	0/280	0/298	0 (0 to 0)	0 (0 to 0)	0 (0 to 0)
	Respiratory distress syndrome requiring oxygen supplementation	10 (3%)/297	4 (1%)/280	6 (2%)/298	2·0 (−0·5 to 4·5)	1·4 (−1·2 to 4·0)	−0·6 (−2·7 to 1·5)
Baby admitted to intensive care unit†		54 (18%)/298	30 (10%)/290	29 (10%)/294	7·8 (2·2 to 13·4)	8·3 (2·7 to 13·8)	0·5 (−4·4 to 5·4)
	Birth asphyxia	1 (<1%)/298	1 (<1%)/290	0/294	0·0 (−0·9 to 0·9)	0·3 (−0·3 to 0·9)	0·3 (−0·3 to 0·9)
	Congenital anomalies	1 (<1%)/298	0/290	0/294	0·3 (−0·3 to 0·9)	0·3 (−0·3 to 0·9)	0 (0 to 0)
	Growth restriction	3 (1%)/298	3 (1%)/290	1 (<1%)/294	0·0 (−1·6 to 1·6)	0·7 (−0·6 to 2·0)	0·7 (−0·6 to 2·0)
	Hypoglycaemia	1 (<1%)/298	0/290	0/294	0·3 (−0·3 to 0·9)	0·3 (−0·3 to 0·9)	0 (0 to 0)
	Low Apgar score	1 (<1%)/298	1 (<1%)/290	0/294	0·0 (−0·9 to 0·9)	0·3 (−0·3 to 0·9)	0·3 (−0·3 to 0·9)
	Low or very low birthweight	37 (12%)/298	17 (6%)/290	20 (7%)/294	6·5 (1·9 to 11·1)	5·6 (0·9 to 10·3)	−0·9 (−4·9 to 3·1)
	Meconium liquor or aspiration	0/298	3 (1%)/290	0/294	−1·0 (−2·1 to 0·1)	0 (0 to 0)	1·0 (−0·1 to 2·1)
	Multiple birth	8 (3%)/298	2 (1%)/290	5 (2%)/294	2·0 (−0·1 to 4·1)	1·0 (−1·4 to 3·4)	−1·0 (−2·8 to 0·8)
	Nasogastric feeding	9 (3%)/298	3 (1%)/290	4 (1%)/294	2·0 (−0·3 to 4·3)	1·6 (−0·8 to 4·0)	−0·4 (−2·2 to 1·4)
	Neonatal convulsion	1 (<1%)/298	3 (1%)/290	1 (<1%)/294	−0·7 (−2·0 to 0·6)	0·0 (−0·9 to 0·9)	0·7 (−0·6 to 2·0)
	Neonatal jaundice	1 (<1%)/298	0/290	1/294 (<1%)	0·3 (−0·3 to 0·9)	0·0 (−0·9 to 0·9)	−0·3 (−0·9 to 0·3)
	Observation in intensive care or greater surveillance by provider	3 (1%)/298	2 (1%)/290	1 (<1%)/294	0·3 (−1·2 to 1·8)	0·7 (−0·6 to 2·0)	0·4 (−0·7 to 1·5)
	Oxygenation	1 (<1%)/298	1 (<1%)/290	1 (<1%)/294	0·0 (−0·9 to 0·9)	0·3 (−0·3 to 0·9)	0·0 (−0·9 to 0·9)
	Preterm	21 (7%)/298	12 (4%)/290	12 (4%)/294	2·9 (−0·8 to 6·6)	2·9 (−0·8 to 6·6)	0·0 (−3·2 to 3·2)
	Refusal to feed	0/298	1 (<1%)/290	0/294	−0·3 (−0·9 to 0·3)	0 (0 to 0)	0·3 (−0·3 to 0·9)
	Respiratory problem or distress	11 (4%)/298	3 (1%)/290	4 (1%)/294	2·7 (0·3 to 5·1)	2·3 (−0·2 to 4·8)	−0·4 (−2·2 to 1·4)
	Sepsis	1 (<1%)/298	4 (1%)/290	0/294	−1·1 (−2·6 to 0·4)	0·3 (−0·3 to 0·9)	1·4 (0·05 to 2·8)
	Small for gestational age	1 (<1%)/298	0/290	0/294	0·3 (−0·3 to 0·9)	0·3 (−0·3 to 0·9)	0 (0 to 0)
	Unknown indication	2 (1%)/298	3 (1%)/290	1 (<1%)/294	−0·3 (−1·8 to 1·2)	0·4 (−0·7 to 1·5)	0·7 (−0·6 to 2·0)
Mean duration of stay in intensive care unit (n=53 *vs* n=29 *vs* n=29)		207·0 (225·5; 0·0–1048·2)	181·7 (171·9; 0·0–609·5)	273·9 (393·9; 0·0–1329·0)	25·2 (−70·5 to 121·0)	−66·9 (−202·8 to 68·9)	−92·2 (−252·0 to 67·7)
	<24 h	6 (11%)/53	7 (24%)/29	2 (7%)/29	−12·8 (−30·6 to 4·9)	4·4 (−8·1 to 17·0)	17·2 (−0·9 to 35·3)
	≥24 h	47 (89%)/53	22 (76%)/29	27 (93%)/29	12·8 (−4·9 to 30·6)	−4·4 (−17·0 to 8·1)	−17·2 (−35·3 to 0·9)
Median duration of stay in intensive care unit, h		111·7 (42·9–266·6)	163·9 (26·6–609·5)	112·7 (64·1–262·0)	−52·2 (−154·8 to 50·3)	−1·0 (−95·7 to 93·7)	51·2 (−58·9 to 161·4)
Baby ventilated		12 (4%)/287	14 (5%)/281	10 (4%)/288	−0·8 (−4·2 to 2·6)	0·7 (−2·4 to 3·8)	1·5 (−1·8 to 4·8)
Neonatal death before discharge†		16 (6%)/287	12 (4%)/283	13 (5%)/288	1·3 (−2·2 to 4·9)	1·1 (−2·5 to 4·6)	−0·3 (−3·6 to 3·1)
	Congenital malformation	0/299	0/290	0/295	0 (0 to 0)	0 (0 to 0)	0 (0 to 0)
	Asphyxia	2 (1%)/299	0/290	3 (1%)/295	0·7 (−0·2 to 1·6)	−0·3 (−1·8 to 1·2)	−1·0 (−2·1 to 0·1)
	Septicaemia	4 (1%)/299	9 (3%)/290	4 (1%)/295	−1·8 (−4·2 to 0·6)	−0·1 (−2·0 −1·8)	1·7 (−0·7 to 4·1)
	Prematurity	12 (4%)/299	7 (2%)/290	10 (3%)/295	1·6 (−1·2 to 4·4)	0·6 (−2·4 to 3·6)	−1·0 (−3·7 to 1·7)
	Low birthweight	2 (1%)/299	1 (<1%)/290	2 (1%)/295	0·4 (−0·7 to 1·5)	0·0 (−1·3 to 1·3)	−0·4 (−1·5 to 0·7)
	Other	7 (2%)/299	1 (<1%)/290	4 (1%)/295	2·0 (0·2 to 3·8)	0·9 (−1·3 to 3·1)	−1·1 (−2·6 to 0·4)

Data are mean (SD; range), n (%), n (%)/N, or median (IQR).

* Outcome unknown because mother was discharged to another facility before delivery.

† More than one indication or cause could be listed.

Seven serious adverse events (in 1% of births) were reported during the study. No birth had more than one adverse event. In addition to the one woman who had an intrapartum seizure, we recorded six (1%) stillbirths within 24 h of the end of study enrolment (one [<1%] stillbirth in the nifedipine group, two [1%] stillbirths in the labetalol group, and three [1%] stillbirths in the methyldopa group). 17 (2%) additional stillbirths occurred more than 24 h after the end of study enrolment and were not reported as severe adverse events. 41 (5%) neonates (16 in the nifedipine group, 12 in the labetalol group, 13 in the methyldopa group) died before discharge; 29 (3%) neonates died because they were premature and 17 (2%) neonates died due to septicaemia. The causes of perinatal death did not differ between groups.

Without use of additional drugs, nifedipine and labetalol use resulted in a greater incidence of the primary outcome than methyldopa use (nifedipine *vs* methyldopa, 19·8% [12·9–26·7]; labetalol *vs* methyldopa, 10·4% [2·9–17·9]; table 3). Women assigned to receive nifedipine were more likely to achieve the blood pressure target at 6 h than those assigned to receive labetalol (p=0·03) or methyldopa (p=0·01). Women assigned to receive nifedipine or labetalol were more likely to reach the blood pressure target without adverse events at 3 h compared with women assigned to receive methyldopa (p=0·002 and p=0·007).

In a prespecified subgroup analysis, we found that women at Daga Women's Hospital (*vs* Government Medical College, Nagpur) were recruited significantly later in pregnancy; had significantly lower systolic and diastolic blood pressure measurements at enrolment; were less likely to have received antihypertensive medication or magnesium sulphate therapy at least 12 h before enrolment or to have abnormal laboratory results at the start of treatment; and were more likely to achieve the primary outcome within 6 h in all three treatment groups (appendix).

Women with a higher systolic or diastolic blood pressure measurements, with evidence of abnormal laboratory results, who had previously received antihypertensive medications, who delivered fetuses at an earlier gestational age, or who were referred into the enrolling centre were less likely to reach the primary outcome within 6 h (appendix). Analysis of the remaining subgroups was uninformative.

The decision to change the sample size after the commencement of the trial resulted in an increased proportion of patients assigned to the methyldopa group later in the study. Thus, we also did a subgroup analysis by phase of enrolment (ie, phase 1 *vs* phase 2). We found that women enrolled in phase 2 of the trial were significantly more likely to achieve the primary outcome within 6 h than women enrolled during the first phase of the trial.

## Discussion

A strength of our study is that it is a direct comparison of the efficacy and safety of the three most commonly used oral antihypertensive medications used for the treatment of severe hypertension in pregnancy. Blood pressure was reduced in a high proportion of women and maternal adverse events were infrequent in all three treatment groups. Oral nifedipine retard was significantly more effective for lowering blood pressure within 6 h than oral methyldopa. However, intensive care unit admissions were significantly more frequent in the nifedipine group. More neonates born to women in the nifedipine group were admitted to the intensive care unit because they had a low or very low birthweight compared with those born to women in the methyldopa or labetalol groups. By contrast, there was no difference in the gestational age at enrolment (table 1) and no difference in the proportion of neonates with birthweights of 1·5 kg or less across the three different treatment groups.

A 2018 network meta-analysis^8^ suggested a similar efficacy of nifedipine, hydralazine, and labetalol in the treatment of severe hypertension in pregnancy. Our results suggest that all three oral medications are viable initial options for treating severe hypertension in pregnancy where additional antihypertensive options are available. Our trial also provides additional information on the relative effectiveness of methyldopa, an oral medication that is widely available in many settings. However, were only a single drug available, nifedipine or labetalol would be preferential to methyldopa. The findings should reassure providers to use the drugs available in their own settings, especially given supply chain and licensing variability between clinical settings.^9^

Our study has some limitations. It was not masked because of the ethical and logistical difficulties in a placebo-controlled trial, which increases the risk of bias. Providers might have had established opinions as to the relative efficacy of different medications, and their opinions could have affected decision making about the administration of an additional dose of the assigned medication or a second medication, especially in the case of methyldopa. Our analysis found that, in most cases, providers complied with the study protocol, and women were not in the target blood pressure range when an additional dose was given. Thus, we believe that the open-label design did not unduly affect the outcomes. The decision to increase the sample size after the start of the trial resulted in an increased proportion of women assigned to the methyldopa group and receiving this treatment in the second half of the trial. Our subgroup analysis found a temporal tend in the data, with women recruited later in the trial (after the change in sample size) more likely to have a blood pressure measurement in the target range. Experience and familiarity with the study protocol could have resulted in greater success as providers gained confidence in the treatment regimens. The effect might have most benefited women assigned to receive methyldopa, who were over-represented in the second phase of the trial.

We found a significant increase in the proportion of admissions to the intensive care unit among neonates born to women assigned to receive nifedipine. The gestational age at delivery, the strongest determinant of birthweight, was similar between groups. Reassuringly, no differences in intubation, survival rates, or lengths of stay among admitted neonates were observed. Although this finding should not be completely discounted, it should probably be the subject of additional investigation before directing care.

We enrolled 894 pregnant women with severe hypertension in low-resource settings. Across the cohort, independent of treatment group, maternal outcomes were very good, and better than expected. There were no maternal deaths, adverse CNS outcomes, maternal admissions for critical care, or need for dialysis. There was only one (<1%) woman with eclampsia, despite use of magnesium sulphate in 12% of women (which halves the risk of eclampsia).^18^ In our opinion, the organised structure for the frequent assessment and prompt management of severe hypertension, including retreatment, to achieve adequate blood pressure control was key. Identifying women with severe hypertension and providing them with available oral antihypertensive drugs in an adequate dose, along with close monitoring for control of blood pressure, established an effective standard for care. Additionally, our trial provided resources to support the protocol while emphasising the role of timely and tight blood pressure control to optimise maternal outcomes. The protocol of treatment and expectation of blood pressure control might be more important to the care of these pregnant women than the differences observed between medications.

Our study suggests the need to expand access to, and use of, oral antihypertensive medications for the treatment of severe hypertension in pregnancy. Given the three-delay model of maternal deaths,^19^ the provision of easily administered oral medications would reduce the burden of the delay in receiving adequate care, from community through to facility. Although WHO has listed only intravenous hydralazine and methyldopa in the latest essential drugs list for treating severe hypertension in pregnancy, in our trial, only one (<1%) woman required an intravenous medication.

## Data sharing

Data collected for the study, including de-identified individual participant data and a data dictionary defining each field in the set, will be made available after publication through contact with the corresponding author. Data will be shared after approval of a proposal by the study authors with a signed data access agreement.
