# Supplementary material for: Oral antihypertensive regimens (nifedipine retard, labetalol, and methyldopa) for management of severe hypertension in pregnancy: an open-label, randomised controlled trial
Source: Lancet. 2019 Sep 21;394(10203):1011–21. doi: 10.1016/S0140-6736(19)31282-6 (PMC6857437; doi:10.1016/S0140-6736(19)31282-6)
Supplement: Supplementary appendix [file mmc1.pdf]

THE LANCET

Supplementary appendix

This appendix formed part of the original submission and has been peer reviewed. We post it as supplied by the authors.

Supplement to: Easterling T, Mundle S, Bracken H, et al. Oral antihypertensive regimens (nifedipine retard, labetalol, and methyldopa) for management of severe hypertension in pregnancy: an open-label, randomised controlled trial. *Lancet* 2019; published online Aug 1. http://dx.doi.org/10.1016/S0140-6736(19)31282-6.

Table A. Side Effects During Study Period (n,%)

	Nifedipine (n=298)	Labetalol (n=295)	Methyldopa (n=301)	Absolute Difference (Nifedipine vs Labetalol) (95% CI)	Absolute Difference (Nifedipine vs Methyldopa) (95% CI)	Absolute Difference (Labetalol vs Methyldopa) (95% CI)
Reported side effect during any monitoring visit during study period						
Nausea	9, 3·0%	11, 3·7%	7, 2·3%	-0·7 (-3·6 – 2·2)	0·7 (-1·9 – 3·3)	1·4 (-1·3 – 4·2)
Vomiting	4, 1·3%	9, 3·1%	3, 1·0%	-1·7 (-4·1 – 0·6)	0·3 (-1·4 – 2·1)	2·1 (-0·2 – 2·1)
RUQ/epigastric pain	0, 0·0%	1, 0·3%	0, 0·0%	-0·3 (-1·0 – 0·3)	0·0 (0·0 – 0·0)	0·3 (-0·3 – 1·0)
Visual disturbance	6, 2·0%	11, 3·7%	5, 1·7%	-1·7 (-4·4 – 1·0)	0·4 (-1·8 – 2·5)	2·1 (-0·5 -4·7)
Chest pain	0, 0·0%	0, 0·0%	2, 0·7%	0·0 (0·0 – 0·0)	-0·7 (-1·6 – 0·3)	-0·7 (-1·6 – 0·3)
Dyspnoea	0, 0·0%	1, 0·3%	0, 0·0%	-0·3 (-1·0 - 0·3)	0·0 (0·0 – 0·0)	0·3 (-0·3 – 1·0)
Vaginal bleeding	0, 0·0%	1, 0·3%	0, 0·0%	-0·3 (-1·0 - 0·3)	0·0 (0·0 – 0·0)	0·3 (-0·3 – 1·0)
Drowsiness	1, 0·3%	2, 0·7%	3, 1·0%	-0·3 (-1·5 – 0·8)	-0·7 (-2·0 – 0·6)	-0·3 (-1·8 – 1·1)
Woman reported headache at monitoring visit						
0h	46, 15·4%	44, 14·9% (n=294)	32, 10·6% (n=300)	0·5 (-5·3 – 6·3)	4·8 (-0·6 – 10·2)	4·3 (-1·1 – 9·6)
2h	46, 15·4% (n=289)	28, 9·5% (n=287)	18, 6·0% (n=295)	5·9 (0·6 – 11·2)	9·4 (4·5 – 14·3)	3·5 (-0·8 – 7·8)
4h	45, 15·6% (n=283)	18, 6·3% (n=282)	15, 5·1% (n=289)	9·3 (4·3 – 14·3)	10·5 (5·6 – 15·4)	1·2 (-2·6 – 4·9)
6h	45, 15·9%	22, 7·8%	10, 3·5%	8·1 (2·8 – 13·4)	12·4 (7·7 – 17·2)	4·3 (0·6 – 8·1)
Mean heart rate (/min)						
0h	96 (55-141)	97 (62-134)	97 (56-142) (n=300)	-1·7 (-3·9 – 0·4)	-1·5 (-3·8 – 0·8)	0·2 (-2·0 – 2·4)
1h	98 (56-139)	94 (54-124) (n=294)	95 (52-142) (n=300)	3·9 (1·9 – 5·9)	0·3 (-5·4 – 5·9)	-3·6 (-9·3 -2·0)
2h	100 (62-138) (n=295)	93 (59-120) (n=292)	94 (54-139) (n=298)	7·0 (5·1 – 9·0)	6·1 (4·0 – 8·3)	-0·9 (-3·0 – 1·2)
3h	100 (63-130) (n=289)	92 (50-119) (n=287)	94 (56-140) (n=295)	7·5 (5·6 – 9·5)	5·9 (3·8 – 8·0)	-1·6 (-3·7 – 0·5)
4h	99 (64-133) (n=285)	92 (59-120) (n=283)	93 (52-132) (n=292)	7·0 (5·1 – 9·0)	6·4 (4·3 – 8·6)	-0·6 (-2·6 – 1·4)
5h	100 (57-150) (n=283)	91 (55-120) (n=282)	93 (54-129) (n=289)	8·3 (6·3 – 10·3)	7·1 (5·0 – 9·2)	-1·2 (-3·2 – 0·9)
6h	98 (67-135)	92 (63-130)	92 (55-128)	6·1 (4·1 – 8·1)	5·9 (3·7 – 8·1)	-0·2 (-2·3 – 1·9)
One or more monitoring visits where pulse >115 (%)	93, 31·2%	42, 14·2%	51, 16·9%	17·0 (10·4 – 23·6)	14·3 (7·5 – 21·0)	-2·7 (-8·5 – 3·1)

Table B. Background characteristics at enrollment (mean (SD) or n, % unless otherwise stated)

	Government Medical College (n=452)	Daga (n=442)
**Maternal demographics and past history**		
Maternal age (years)	25·7 (4·1)	25·3 (4·1)
BMI	25·6 (4·0) (16·2-38·0)	29·0 (3·9) (19·1-43·7)
**Pregnancy characteristics**		
Gestational age (weeks)	35·4 (2·6)	37·8 (2·4)
Multiple pregnancy	18, 4·0%	10, 2·3%
Fetus alive at enrollment (n, %)	298, 100%	295, 100%
BP measurements		
Mean systolic BP	162 (12·0) (130-210)	157 (10·4) (130-190)
Highest systolic BP at enrollment (n, %)*		
< 160 mmHg	125, 27·7%	195, 44·1%
160-169 mmHg	193, 42·7%	173, 39·1%
≥170 mmHg	134, 29·6%	74, 16·7%
Mean diastolic BP	110 (7·5) (90-140)	110 (6·0) (90-130)
Highest diastolic BP at enrollment (mmHg) (n, %)		
< 110	103, 22·8%	90, 20·4%
110-119	276, 61·1%	311, 70·4%
≥120	73, 16·2%	41, 9·3%
Urinary dipstick proteinuria		
Nil or Trace	164, 36·3%	147, 33·3%
+1	127, 28·1%	161, 36·4%
+2	98, 21·7%	101, 22·9%
>+3	63, 13·9%	33, 7·5%
Oxygen saturation <95% (n,%)	1, 0·2%	0, 0·0%
Mean HR at 0h (SD)(range)	91·5 (13·8) (55-140)	102·1 (11·5) (65-142)
Source of admission (n,%)		
Referred	217, 48·0%	45, 10·2%
Outpatient department	231, 51·1%	301, 68·1%
Walk-in	4, 0·9%	96, 21·7%
Received MgSO4 in 12h preceding enrollment (n,%)	36, 8·0%	2, 0·5%
Received antihypertensive drug in 12 or more hours before enrollment	184, 40·7%	5, 1·3%
Methyldopa	38, 8·4%	3, 0·7%
Nifedipine	93, 20·6%	1, 0·2%
Labetalol	61, 13·5%	2, 0·5%
Plans for delivery at time of enrolment		
Planned induction of labor	338, 74·7%	24, 5·4%
Planned expectant management	114, 25·2%	418, 94·6%
**Laboratory values**		
Platelet <100k (n,%)	38/448, 8·5%	7/420, 1·2%
% with serum creatinine ≥1.0	55/450, 12·2%	10/438, 2·3%
AST more than 2x normal (>80 IU/L) (n,%)	42/450, 9·3%	0/437, 0·0%
AST more than 2x normal (>80 IU/L) and platelets <100,k at 0h	5/448, 1·1%	0/417, 0·0%

Table C. Estimated odds ratios, adjusted odds ratios and 95% confidence intervals for factors associated with achievement of primary outcome* (n=894)

Variable				
	**Odds Ratio**	**95% CI**	**Adjusted Odds Ratio**	**95% CI**
Treatment				
Nifedipine	1.00		1.00	
Labetalol	0.67	0.44-1.01	**0.61**	**0.38-0.97**
Methyldopa	**0.64**	**0.42-0.96**	**0.43**	**0.27-0.68**
Study site				
Government Medical College	1.00		1.00	
Daga	**3.90**	**2.71-5.62**	**2.36**	**1.37-4.08**
Received antihypertensive drug 12h before enrollment				
No	1.00		1.00	
Yes	**0.36**	**0.25-0.52**	**0.61**	**0.39-0.96**
Systolic BP at 0h (continuous variable)	**0.95**	**0.94-0.96**	**0.96**	**0.95-0.98**
Diastolic BP at 0h (continuous variable)	**0.96**	**0.94-0.99**	**0.97**	**0.95-0.99**
Pulse rate at 0h (continuous variable)	**1.02**	**1.01-1.03**	1.00	0.98-1.01
Phase of enrollment†				
Phase 1	1.00		1.00	
Phase 2	**1.51**	**1.08-2.13**	**1.79**	**1.18-2.72**
Mean arterial pressure at 0h (continuous variable)	**0.92**	**0.90-0.94**		
Mode of admission				
Referred	1.00		1.00	
OPD	**2.06**	**1.46-2.92**	0.98	0.65-1.49
Walk-in	**2.27**	**1.25-4.11**	0.64	0.30-1.33
Evidence of abnormal laboratory results‡				
Normal	1.00		1.00	
Abnormal	**0.38**	**0.26-0.57**	0.68	0.42-1.08
Yes				
Gestational age (continuous variable)	**1.16**	**1.09-1.23**	1.04	0.96-1.12

*Primary outcome: reached the BP target (defined as 120-150 mmHg systolic AND 70-100 mmHg diastolic) at 6h without an adverse outcome occurring during the study period (i.e. systolic BP<120 and/or diastolic <70 and fetal compromise; caesarean section for fetal distress, severe headache or eclampsia)

† Phase 1: randomization with original sample size; Phase 2: randomization with increased sample size

‡ Definition of abnormal lab results: Platelets <100,000 OR AST >2x normal OR serum creatinine > 1.0

§ Number of participants was too low for analysis by history of chronic hypertension (n=2) or diabetes or gestational diabetes in this pregnancy (n=2).

Abbreviations: Odds ratio by univariate logistic regression. 95% CI, 95% confidence interval. Adjusted odds ratio by multiple logistic regression adjusted for treatment group, site, receipt of antihypertensive medication 12 or more hours prior to enrollment, systolic BP at 0h, diastolic BP at 0h, heartrate at 0h, and randomization scheme (Phase 1 vs. Phase 2). Bold type indicates that the CI does not include the null according to logistic regression analyses.
